# Carbon ion radiotherapy for oligo-recurrent lung metastases from colorectal cancer: a feasibility study

**DOI:** 10.1186/1748-717X-9-68

**Published:** 2014-03-01

**Authors:** Wataru Takahashi, Mio Nakajima, Naoyoshi Yamamoto, Shigeru Yamada, Hideomi Yamashita, Keiichi Nakagawa, Hiroshi Tsuji, Tadashi Kamada

**Affiliations:** 1 Research Center Hospital for Charged Particle Therapy, National Institute of Radiological Sciences, 4-9-1 Anagawa, Inage-ku, Chiba-shi, Chiba 263-8555, Japan; 2 Department of Radiology, The University of Tokyo Hospital, 7-3-1 Hongo, Bunkyo-ku, Tokyo 113-8655, Japan

**Keywords:** Carbon ion radiotherapy, Colorectal cancer, Lung metastases, Prognosis, Oligometastases, Local control, Survival, Toxicity

## Abstract

**Background:**

The purpose of this study was to evaluate the efficacy and feasibility of carbon ion radiotherapy (CIRT) for oligo-recurrent lung tumors from colorectal cancer (CRC).

**Methods:**

From May 1997 to October 2012, 34 consecutive patients with oligo-recurrent pulmonary metastases from CRC were treated with CIRT. The patients were not surgical candidates for medical reasons or patient refusal. Using a respiratory-gated technique, carbon ion therapy was delivered with curative intent using 4 coplanar beam angles. A median dose of 60 GyE (range, 44–64.8 GyE) was delivered to the planning target volume (PTV), with a median daily dose of 15 GyE (range, 3.6–44 GyE). Treatment outcome was analyzed in terms of local control rate (LCR), survival rate, and treatment-related complications.

**Results:**

In total, 34 patients with 44 oligo-recurrent pulmonary lesions were treated with CIRT. Median follow-up period was 23.7 months. The 2- and 3-year actuarial LCRs of the treated patients were 85.4% ± 6.2% and 85.4% ± 6.2%, respectively. Overall survival was 65.1% ± 9.5% at 2 years, and 50.1% ± 10.5% at 3 years. Although survival rates were relatively worse in the subsets of patients aged < 63 years or with early metastasis (< 36 months after resection of primary site), these factors were not significantly correlated with overall survival (*P* = 0.13 and 0.19, respectively). All treatment-related complications were self-limited, without any grade 3–5 toxicity.

**Conclusions:**

CIRT is one of the most effective nonsurgical treatments for colorectal lung metastases, which are relatively resistant to stereotactic body radiotherapy. CIRT is considered to be the least invasive approach even in patients who have undergone repeated prior thoracic metastasectomies.

## Background

Colorectal cancer (CRC) is the third most commonly diagnosed cancer in the world [1]. The lung is a frequent site for metastasis after definitive therapy of CRC, with pulmonary metastases occurring in approximately 10% of patients [2,3]. The presence of pulmonary metastases was previously assumed to represent disseminated incurable disease, and palliative chemotherapy was usually recommended. Recent innovations in diagnostic imaging, such as positron emission tomography/computed tomography (PET/CT), allow the detection of small numbers of metastases, usually confined to one or two locations. Niibe and Hayakawa proposed the term “oligo-recurrence” to describe a few metastases with a controlled primary site and “oligometastases” to describe a similar small number of metastases with the primary tumor still present. They stated that, in these cases (particularly in oligo-recurrence), aggressive local therapy could result in prolonged disease-free survival with the possibility of cure [4].

For cases with oligo-recurrent pulmonary lesions, metastasectomy has been considered standard management, yielding a five-year survival rate of up to 60% [3,5-7]. However, a significant proportion of these patients are not candidates for metastasectomy due to their advanced age and/or coexistent morbidities such as low pulmonary function. Similar to strategies in early-stage non-small cell lung cancer (NSCLC), stereotactic body radiotherapy (SBRT) and particle radiotherapy, such as carbon ion radiotherapy (CIRT), have emerged as new treatment options for metastatic carcinoma [8-15]. Although many studies have reported favorable outcomes with SBRT for pulmonary metastases [8-13,15], Takeda et al. reported that local control after SBRT for lung metastases from CRC is worse than that from other origins, with a 2-year LCR of 72% vs 94%, respectively (*P* < 0.05) [11]. Takahashi et al. have also demonstrated relatively poor local control of oligo-recurrent pulmonary lesions in CRC patients undergoing lung SBRT [13].

In our previous study, we investigated the use of CIRT as a single modality in oligo-recurrent pulmonary metastases in patients who were not surgical candidates by evaluating clinical outcomes of CIRT in oligo-recurrent pulmonary lesions from various primary sites. This is the only study of CIRT in the management of oligo-recurrent pulmonary lesions reported to date [14].

Here, we analyze the outcomes in the CRC subpopulation of that study over a longer period of follow-up. To our knowledge, this study is the first assessment of CIRT for oligo-recurrent pulmonary metastases from CRC.

## Methods

### Patient eligibility and pretreatment evaluation

We conducted a retrospective review of 34 consecutive patients with oligo-recurrent pulmonary metastases from CRC treated with CIRT at the National Institute of Radiological Sciences (NIRS) from May 1997 to October 2012. All patients had already undergone radical surgery for the primary site, which was histologically proven to be colorectal adenocarcinoma. During follow-up, they developed oligo-recurrent pulmonary lesions and were not candidates for surgical resection for medical reasons or patient refusal. The histology and diagnosis of the metastatic tumors were determined based on the imaging findings and clinical course. Pretreatment evaluation included a complete medical history, physical examination, chest radiography, computed tomography (CT) of the chest and abdomen, contrast-enhanced brain magnetic resonance imaging (MRI), PET/CT scanning, electrocardiography, pulmonary function tests, and blood tests. Inclusion criteria for the current study were as follows: (a) the primary cancer was completely resected, with no evidence of recurrence; (b) the number of metastases was only one at the time of treatment; (c) no other distant metastasis in organs other than the lungs; (d) no systemic therapy such as chemotherapy within 1 month of initiation of CIRT; (e) life expectancy of greater than 6 months, plus adequate pulmonary function. There were no exclusions regarding tumor size or location in the lung. Patients developing additional metastases during follow-up were not excluded. All patients provided written informed consent before undergoing CIRT. This study was approved by the institutional review board at our institution (NIRS-#0504G protocol).

### CIRT planning and dose delivery

Details of CIRT planning and delivery at our institution have been described previously [14,16,17]. Briefly, patients were immobilized in the supine or prone position with a custom-made device without an abdominal compressor. Planning CT images were then acquired with 2.5- or 5.0-mm-thick slices using a 16-slice CT scanner. These data were then sent to a three-dimensional radiation treatment planning system (3D RTPS; HIPLAN, software developed at NIRS) and respiratory-gated CIRT plans were created by calculating dose distribution with a parallel broad beam algorithm. Carbon ion beams of 250 or 290 MeV, depending on the target size and water equivalent path length (WEPL) along the beam line, were generated by the Heavy Ion Medical Accelerator in Chiba (HIMAC) synchrotron.

**Figure 1:**
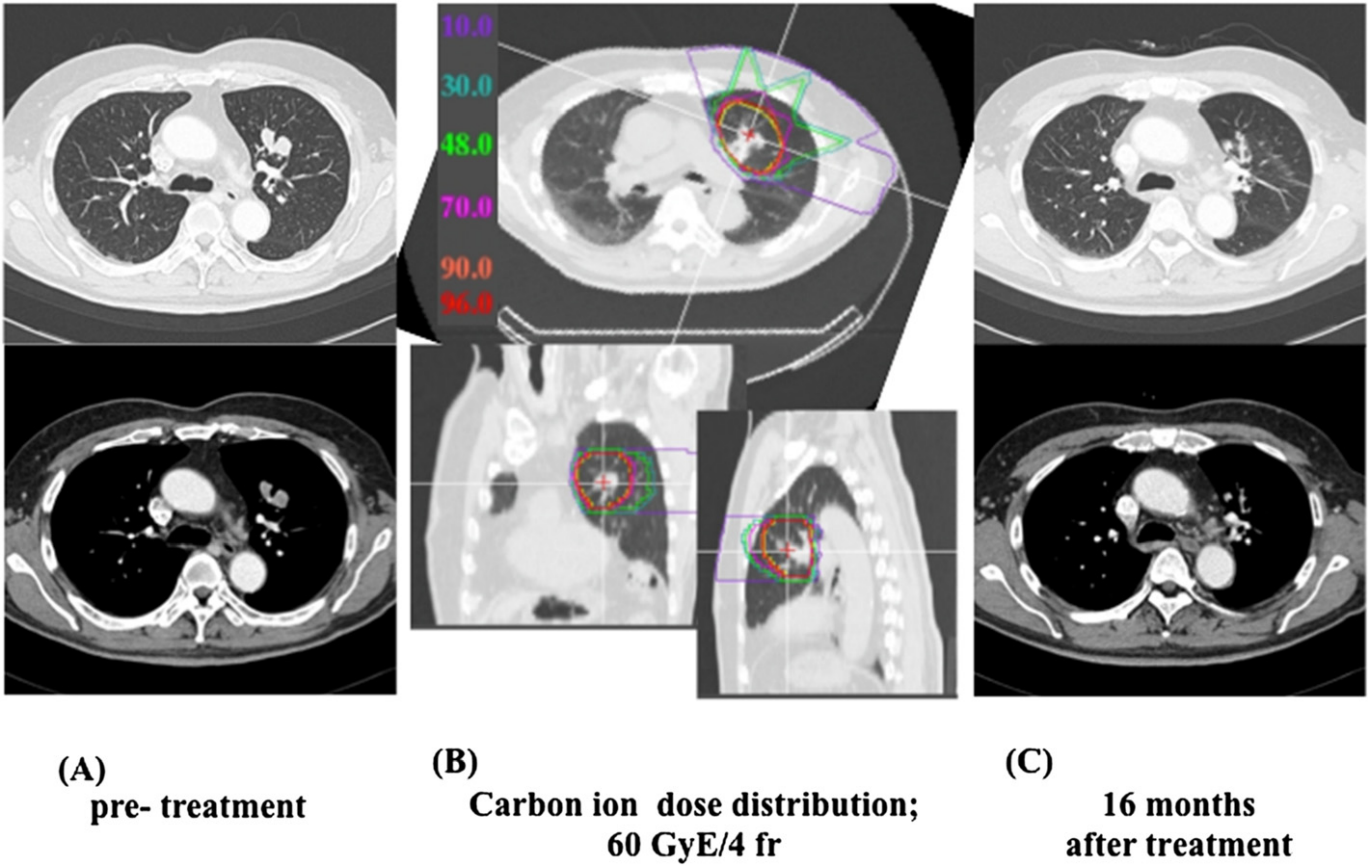
**Carbon ion radiotherapy and radiographic changes in a typical patient. (A)** Pre-treatment CT scan of oligo-recurrence in the lung from colorectal cancer. **(B)** Radiotherapy plan for oligo-recurrent pulmonary lesion, which received 60 GyE in four fractions. The yellow line indicates the clinical target volume that was delineated in the expiratory phase. Dose distribution is shown with isodose lines of different colors (color scale on left side of image). The clinical target volume is fully encompassed by the 95% isodose line. **(C)** Chest CT scan 16 months after irradiation, revealing no evidence of recurrence.

Gross tumor volume (GTV) was manually delineated in lung windows in the expiratory phase. Clinical target volume (CTV) was created by adding over 10 mm margins to the GTV in all directions. To allow for the intrafractional motion of the tumor during gated irradiation, a 5-mm internal margin (IM) was added to the CTV in the craniocaudal direction. Then, the planning target volume (PTV) was defined as the CTV plus this IM and a 5-mm safety margin for positioning uncertainty. In accordance with our routine practice for Stage I NSCLC, all CIRT plans utilized 4 coplanar and oblique beam fields at a mutual angle of 40 or 50 degrees. The total dose was applied to the isocenter, and the PTV was enclosed conformally at the minimum by the 95% isodose line with the prescribed dose. CIRT was delivered at a median total dose of 60 GyE (range, 44–64.8 GyE) with curative intent. The prescribed dose and fractionation schedules were determined by reference to the Stage I NSCLC treatment protocol of the period. A fractionation regimen of 60.0 GyE in 4 fractions was the schedule most commonly used for the treatment of 31 of 44 total lesions. Figure 1 shows an example of contours and isodose curves for a typical case.

### Follow-up, evaluation and statistics

After completion of therapy, clinical follow-up included clinical examination, chest CT, and laboratory tests every 3 months for the first 2 years and every 6 months thereafter. Additional imaging investigations, such as PET/CT, were ordered if there was clinical suspicion of recurrence. Treatment outcomes were analyzed in terms of local control rate, survival rate, and treatment-related complications. In this study, local recurrence was defined as a continuous increase in opacity size on CT imaging, along with either increased maximum standardized uptake values (SUVmax) ≥ 5 on PET/CT, or biopsy proof of disease [18].

Skin and lung toxicities were assessed according to the National Cancer Institute Common Terminology Criteria for Adverse Events (NCI CTCAE) version 3.0, with toxicity occurring within 3 months after the initiation of CIRT classified as acute toxicity. Late toxicity was graded using the Radiation Therapy Oncology Group (RTOG)/European Organisation for Research and Treatment of Cancer (EORTC) criteria.

All data are presented as median values with ranges. Overall survival (OS) was defined as the elapsed time from the start of CIRT to death or to the last follow-up date (the last follow-up date was the last hospital visit, phone, or mail day). Patients lost to follow-up or alive at the end of the study were considered censored. LCR was calculated from the initial CIRT date to the first local recurrence date or the date of the patient’s last visit. OS and LCR were analyzed using Kaplan-Meier statistics.

Sex, age (≥ 63 vs. < 63 years), primary tumor site (colon vs. rectum), history of previous systemic chemotherapy, past history of pulmonary resection for previous metastases, disease-free interval (DFI, meaning interval between the date of initial surgery for primary site and start date of CIRT; ≥ 36 months vs. < 36 months), pretreatment carcinoembryonic antigen (CEA) level, and maximum diameter of lung tumor (≥ 18 mm vs. < 18 mm) were analyzed using the log-rank test. The thresholds of age, DFI, and diameter, were chosen based on the median of each value over all patients. A *P*-value less than 0.05 was defined as statistically significant.

## Results

### Patient characteristics

In our database, we identified 34 patients with 44 oligo-recurrent pulmonary lesions treated with CIRT. Patient and tumor characteristics are summarized in Table 1. There were 20 men (59%) and 14 women (41%), and age ranged from 34 to 79 years with a median of 63 years at the time of therapy. Karnofsky performance status (K-PS) of these patients was relatively robust; 2 were 80% (5.9%), 21 were 90% (61.8%), and 11 were 100% (32.4%). The primary CRC was located in the colon in 15 patients and in the rectum in 19. Eighteen (53%) patients had received ≥ 1 chemotherapy regimen before CIRT, and 10 (29%) had undergone previous pulmonary metastasectomy for other oligo-recurrent pulmonary lesions. Median CEA before pulmonary CIRT was 3.5 μg/L (range, 0.5–650.0 μg/L). The median maximum diameter of metastatic tumors was 18.0 mm (range, 5.0–60.0 mm), and CTV ranged from 11.4-165.4 mL with a median of 51.7 mL.

**Table 1:** Summary of patient and tumor characteristics

**Variables**	**Distribution**		**(%)**
Total patients		34	
Total lesions		44	
Gender			
	Male	20	(59)
	Female	14	(41)
Age (years)			
	Median	63	
	Range	34-79	
Primary cancer			
	Colon	15	(44)
	Rectum	19	(56)
Previous systemic chemotherapy			
	Yes	18	(53)
	No	16	(47)
Previous pulmonary resection			
	Yes	10	(29)
	No	24	(71)
Disease-free interval until lung metastasis (months)			
	Median	46.3	
	Range	10.8-100.9	
Carcinoembryonic antigen (CEA) before CIRT			
	Median	3.5	
	Range	0.5-650.0	
Number of lesions treated by CIRT (per patient)			
	1	28	
	2	4	
	3	0	
	4	2	
Maximum diameter of lung tumor (mm)			
	Median	18.0	
	Range	5.0-60.0	
Tumor location (lobe)			
	Left upper/lower	17/5	
	Right upper/middle/lower	10/3/9	
Fractionation sheme			
	60 GyE/4 fr	31	
	52.8 GyE/4 fr	6	
	64.8 GyE/12 fr	2	
	57.6 GyE/16 fr	1	
	60 GyE/12 fr	1	
	60 GyE/8 fr	1	
	48 GyE/8 fr	1	
	44 GyE/1 fr	1	
Follow-up (months)			
	Median	23.7	
	Range	6.1-167.0	

*Abbreviations*: *CIRT* carbon ion radiotherapy.

After initial CIRT, 6 patients in whom new additional single oligo-recurrent pulmonary lesions occurred were treated with further CIRT in our institution according to the abovementioned criteria. In total, 4 lesions were irradiated in 2 patients, 2 lesions were treated in 4 patients, and only one lesion was treated in 28 patients.

### Local control and survival

**Figure 2:**
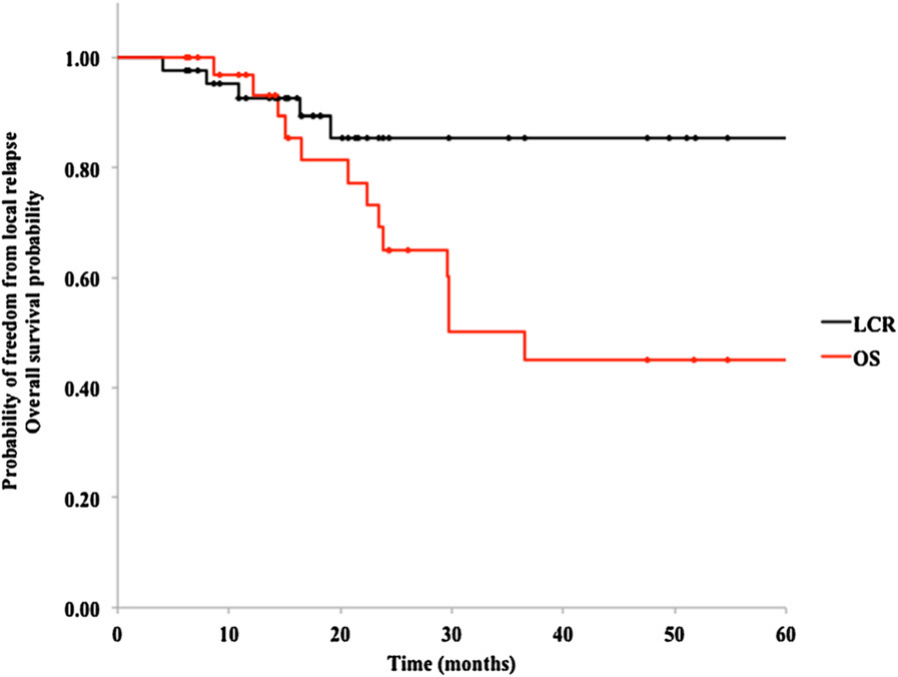
Overall survival and local control in 34 patients with oligo-recurrent metastatic disease in the lung.

Median follow-up duration was 23.7 months (range, 6.1 to 167.0 months) in the entire population and 24.4 months (range, 6.1 to 167.0 months) for those alive. At the time of last follow-up, 13 patients had died and 16 patients were disease-free. The 2- and 3-year LCR of the treated patients were 85.4% (95% CI, 73.2–97.6%) and 85.4% (95% CI, 73.2%–97.6%), respectively (Figure 2), and median time to local recurrence was 10.8 months. Except for one patient who died of bacterial pneumonitis, all deaths were due to local CRC recurrence or metastases (all extrahepatic). The overall 2- and 3-year survival rates were 65.1% (95% CI, 46.5%–83.6%) and 50.1% (95% CI, 29.4%–70.7%), respectively (Figure 2), with a median survival time of 36.6 months. Eleven of 34 patients (32.4%) showed a long-term survival of longer than 3 years.

**Figure 3:**
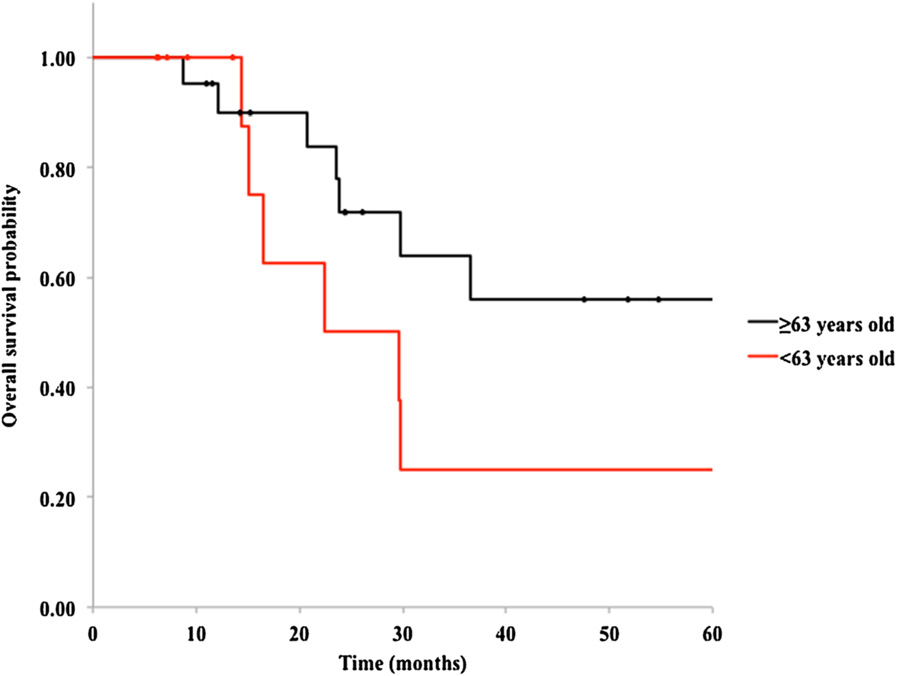
**Kaplan-Meier curves for overall survival in 34 patients with oligo-recurrent metastatic disease in the lung; younger age (< 63 years old) and older age (≥ 63 years old) (*P*** **= 0.13).**

**Figure 4:**
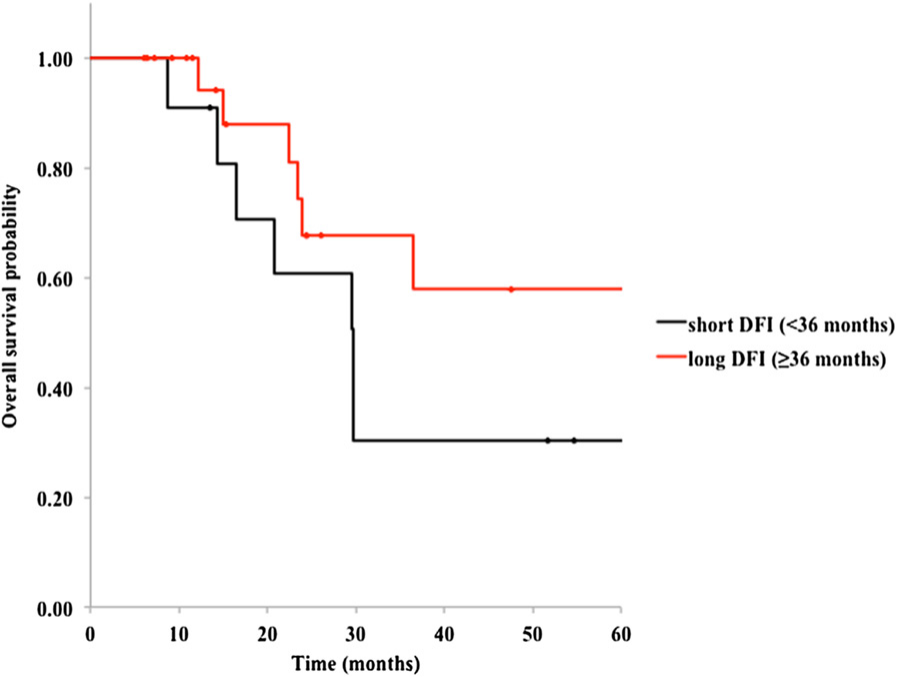
**Kaplan-Meier curves for overall survival in 34 patients with oligo-recurrent metastatic disease in the lung; early metastases subgroup versus late metastases subgroup (*P*** **= 0.19).**

We also analyzed differences in OS on stratification by various factors. As shown in Figures 3 and 4, patients aged younger than 63 and those with early metastasis (short DFI) had relatively worse prognoses than those with late recurrence, but these differences were not statistically significant (*P* = 0.13 and 0.19, respectively). Other factors, including sex, primary site, a history of chemotherapy or metastasectomy, CEA level before CIRT, and tumor size, were not significantly associated with survival (Table 2).

**Table 2:** Assessment of prognostic factors for overall survival

**Factor**	**Group**	**No. of patients**	**3-year overall surviaval**	**Log-rank test P value**
Sex	Male	20	62.9%	0.47
	Female	14	36.9%	
Age (years)	≥63	21	64.0%	0.13
	<63	13	25.0%	
Primary cancer	Colon	15	53.9%	0.87
	Rectum	19	44.6%	
Previous systematic chemotherapy	Yes	18	39.6%	0.79
	No	16	56.8%	
Previous pulmonary resection	No	10	47.6%	0.60
	Yes	24	68.1%	
Interval between initial surgery and first CIRT	≥36 month	23	67.6%	0.19
	<36 months	11	30.3%	
CEA before first CIRT	<5	20	42.9%	0.98
	≥5	14	68.1%	
Maximun diameter of lung tumor	≥18 mm	21	51.8%	0.60
	<18 mm	13	50.0%	

*Abbreviations*: *CEA* carcinoembryonic, *CIRT* carbon ion radiotherapy.

### Adverse events

All patients completed their CIRT course without interruption. The most common acute toxicities included radiation pneumonitis (RP) and dermatitis. Of the 34 patients, 28 patients (82%) and 5 patients (15%) experienced grade 1 (asymptomatic, radiographic findings only) and grade 2 (symptomatic, not interfering with activities of daily living) RP, respectively. Transient mild erythema (grade 1–2) occurred in most patients. No case of grade 3–5 acute or late toxicity has been recorded up to this writing.

## Discussion

Based on the suggestion that patients with oligo-recurrence experience better survival, various non-surgical modalities have been used as local treatment for the management of inoperable oligo-recurrent pulmonary lesions. Many series have shown that SBRT provides good local control in ablating lung metastases [8-13,15]. However, given the relative radioresistance of CRC metastases, dose escalation would be needed to achieve the same results. Norihisa et al. proposed dose escalation in SBRT for oligo-recurrent pulmonary metastases to achieve better local control [8], but identification of grade 3–5 toxicity in up to 15% of patients indicates the limitations of raising the dose [19]. Radiofrequency ablation (RFA) is known to provide effective local therapy for metastatic lung tumors. Petre and colleagues recently reported the results of RFA in the treatment of 69 oligo-recurrent pulmonary lesions in 45 CRC patients. Without any serious adverse events, 2- and 3-year local control rates of 77% were promising and similar to those with SBRT [20]. Although RFA is safer than surgical resection and effective for unresectable oligo-recurrent pulmonary lesions, iatrogenic pneumothorax is estimated to occur in 30% to 40% of patients, and about 35% of this subgroup require treatment with chest tube placement [20-22].

We report our experience with a series of 34 patients treated with CIRT for oligo-recurrent pulmonary metastases from CRC. With a median follow-up of 23.7 months, the 2-year actuarial LCR and OS were 85.4% and 65.1%, respectively. CIRT promises good local control of metastatic CRC and survival comparable to rates achieved with surgical resection. Despite the medical comorbidities and advanced age of most patients, it should be noted that all the patients were treated safely. All treatment-related complications were self-limited, without any grade 3–5 toxicity. We consider that CIRT is the least invasive approach to oligo-recurrent pulmonary metastases, even in patients who have undergone repeated prior pulmonary metastasectomies. CIRT has the advantage of delivering a high linear energy transfer (LET) radiation dose to the target with sparing of uninvolved lung parenchyma and surrounding critical organs. These characteristics of CIRT may improve the LCR while diminishing the likelihood of adverse events.

Salvage CIRT for the relapsing lesion after SBRT has the potential benefit of high dose delivery in a short treatment time with markedly low toxicity. This is important topic, but is beyond the scope of this study and will be evaluated in our future study. In fact, some local-relapse cases after SBRT have already treated using CIRT and achieved local control in our institution. However, no recurrent patients underwent SBRT was included in this study.

Our analysis of the OS differences stratified by DFI (Figure 4) showed a nonsignificant trend of shorter DFI leading to poorer prognosis (*P* = 0.19). Both in surgical resection and nonsurgical treatments such as SBRT, several investigators reported significantly higher OS for patients with a DFI of greater than 3 years [3,5,8,21].

### Limitations

Our study has several limitations. First, the study was retrospective in its data collection, with a relatively short follow-up time. Additional follow-up to assess the long-term impact of CIRT on clinical outcomes is necessary. No histology was obtained on the lung lesions, some of which may have been more radiosensitive NSCLCs, which would have resulted in the overestimation of treatment effect. Given that all lesions had the typical imaging appearance of metastatic lung tumors and grew rapidly during follow-up after primary resection, however, we consider that the possibility of misdiagnosis was low. Another limitation is the various dose treatment protocols using a wide range of dose prescriptions and variety of fractionation schemes. Given the very good clinical outcomes, however, CIRT warrants consideration as a nonsurgical alternative for the management of oligo-recurrent pulmonary lesions. We recommend the current standard CIRT regimen of 60 GyE in 4 fractions, the dose delivered to the majority of our cohort.

## Conclusions

Our results indicate that CIRT is one of the most effective modalities for treatment of oligo-recurrent pulmonary metastases from CRC, which are relatively resistant to SBRT. Similar to its use in early-stage NSCLC, CIRT did not induce severe complications, even in elderly patients who were not surgical candidates. We suggest that the best candidates for CIRT are patients with controlled primary CRC tumors and a single oligometastatic site in the lung.

## Abbreviations

CIRT: Carbon ion radiotherapy; CRC: Colorectal cancer; PTV: Planning target volume; LCR: Local control rate; PET/CT: Positron emission tomography/computed tomography; NSCLC: Non-small cell lung cancer; SBRT: Stereotactic body radiotherapy; NIRS: National institute of radiological sciences; MRI: Magnetic resonance imaging; 3D RTPS: Three-dimensional radiation treatment planning system; WEPL: Water equivalent path length; HIMAC: Heavy ion medical accelerator in Chiba; GTV: Gross tumor volume; SUVmax: Maximum standardized uptake value; NCI CTCAE: National cancer institute common terminology criteria for adverse events; RTOG/EORTC: Radiation therapy oncology group/European organisation for research and treatment of cancer; OS: Overall survival; DFI: Disease-free interval; CEA: Carcinoembryonic antigen; K-PS: Karnofsky performance status; RP: Radiation pneumonitis; RFA: Radiofrequency ablation; LET: Linear energy transfer.

## Competing interests

The authors declare that they have no conflicts of interest.

## Authors’ contributions

WT collected and analyzed data and performed statistical analysis. WT and NY drafted the manuscript. MN, HT, HY and KN reviewed the data and revised the manuscript. NY, SY and TK designed the study and revised the final version. All authors read and approved the final manuscript.
